# Stress-sensing in the human greying hair follicle: Ataxia Telangiectasia Mutated (ATM) depletion in hair bulb melanocytes in canities-prone scalp

**DOI:** 10.1038/s41598-020-75334-9

**Published:** 2020-10-30

**Authors:** Stephen K. Sikkink, Solene Mine, Olga Freis, Louis Danoux, Desmond J. Tobin

**Affiliations:** 1 Centre for Skin Sciences, School of Life Sciences, University of Bradford, Richmond Rd., Bradford, BD7 1DP West Yorkshire UK; 2 BASF Beauty Care Solutions France S.A.S., Pulnoy, France; 3 The Charles Institute of Dermatology, School of Medicine, University College Dublin, Dublin 4, Ireland

**Keywords:** Cellular imaging, Predictive markers

## Abstract

Canities (or hair greying) is an age-linked loss of the natural pigment called melanin from hair. While the specific cause(s) underlying the loss of melanogenically-active melanocytes from the anagen hair bulbs of affected human scalp remains unclear, oxidative stress sensing appears to be a key factor involved. In this study, we examined the follicular melanin unit in variably pigmented follicles from the aging human scalp of healthy individuals (22–70 years). Over 20 markers were selected within the following categories: melanocyte-specific, apoptosis, cell cycle, DNA repair/damage, senescence and oxidative stress. As expected, a reduction in melanocyte-specific markers in proportion to the extent of canities was observed. A major finding of our study was the intense and highly specific nuclear expression of Ataxia Telangiectasia Mutated (ATM) protein within melanocytes in anagen hair follicle bulbs. ATM is a serine/threonine protein kinase that is recruited and activated by DNA double-strand breaks and functions as an important sensor of reactive oxygen species (ROS) in human cells. The incidence and expression level of ATM correlated with pigmentary status in canities-affected hair follicles. Moreover, increased staining of the redox-associated markers 8-OHdG, GADD45 and GP-1 were also detected within isolated bulbar melanocytes, although this change was not clearly associated with donor age or canities extent. Surprisingly, we were unable to detect any specific change in the expression of other markers of oxidative stress, senescence or DNA damage/repair in the canities-affected melanocytes compared to surrounding bulbar keratinocytes. By contrast, several markers showed distinct expression of markers for oxidative stress and apoptosis/differentiation in the inner root sheath (IRS) as well as other parts of the hair follicle. Using our in vitro model of primary human scalp hair follicle melanocytes, we showed that ATM expression increased after incubation with the pro-oxidant hydrogen peroxide (H_2_O_2_). In addition, this ATM increase was prevented by pre-incubation of cells with antioxidants. The relationship between ATM and redox stress sensing was further evidenced as we observed that the inhibition of ATM expression by chemical inhibition promoted the loss of melanocyte viability induced by oxidative stress. Taken together these new findings illustrate the key role of ATM in the protection of human hair follicle melanocytes from oxidative stress/damage within the human scalp hair bulb. In conclusion, these results highlight the remarkable complexity and role of redox sensing in the status of human hair follicle growth, differentiation and pigmentation.

## Introduction

Canities or hair greying is associated with the aging process and characterised by new growth of unpigmented hair fibre within hair follicles^1^. Under normal conditions hair follicle melanocytes actively produce and transfer melanin to pre-cortical keratinocytes during the anagen growth phase of the hair follicle cycle, leading to a fully pigmented hair fibre. During subsequent rounds of hair cycling it is thought that transient amplifying melanocytes, derived from a pool of stem-cell like melanoblasts located in the bulge region (niche), repopulate and pigment the newly formed hair follicle^2^. Work by Nishimura and colleagues confirmed the location of the melanocyte stem cell niche in murine hair follicles to the lower portion of the bulge region where melanoblasts can repeatedly migrate out of, divide and repopulate the niche again^3–6^. Once these cells move to the hair bulb they differentiate into mature melanocytes and transfer pigment into the cortex keratinocytes. Despite extensive studies of the biochemical and cellular pathways that influence pigmentation, the exact molecular mechanisms that lead to hair greying in the human anagen hair follicle are still relatively unclear^7,8^. Studies (albeit predominantly in mice) have pointed to a number of factors including increased oxidative stress in the hair follicle^9^, depletion of melanoblast stem cells from the niche^10^, apoptosis of differentiated matrix melanocyte in the hair bulb^11^ and DNA damage repair (DDR) abnormalities, that all may contribute in part to hair greying^12^. The reader is directed to an excellent recent review on the topic by O’Sullivan and colleagues^13^.

Oxidative stress is caused by an imbalance in pro- and anti-oxidant systems in cells and tissues and can increase damage to all biomolecules including proteins and DNA^14–17^. Uncontrolled levels of reactive oxygen species (ROS) have been implicated in aging^18^ and in a variety of pathological conditions like dementia^19,20^ and various cancers^21–23^. In melanocytes, ROS can be produced from external sources such as chemical/mechanical environmental stressors^24^ or through intrinsic cellular processes such as melanogenesis in response to ultra-violet (UV) radiation^25–28^. Usually cells (including melanocytes) have distinct overlapping mechanisms for coping with ROS/antioxidant imbalance from multiple sources to prevent cellular damage^29–33^. The transcription factor nuclear erythroid 2-related factor (NRF2) is a major component of the cells response to oxidative stress^34–36^ and is expressed at very high levels in epidermal cells^37^. NRF2 provides a protective effect in melanocytes in response to both UV irradiation and oxidative stress^30,31^, through transcriptional upregulation of the antioxidant response element (ARE) genes including Superoxide dismutase-1 (SOD-1), Glutathione peroxidase-1 (GP-1), Glutathione reductase-1 (GR-1), Catalase and Heme oxygenase-1 (HO-1)^38–40^. Studies in melanocytes have demonstrated that levels of HO-1 and its interaction with NFR2 are quickly upregulated in response to ROS^30,31,41^. A study by Haslam, et al*.,* in human ex vivo hair follicles, also showed that NRF2 was crucial during H_2_O_2_ induced oxidative stress in co-ordinating specific anti-oxidant response elements including GR-1 and HO-1^42^.

Aside from antioxidant metabolism and regulation in response to elevated ROS, cells also recruit components of the DNA damage response (DDR) pathway to cope with the potential damaging effect of free radicals on DNA^29,43^. A central component in this response is the *Ataxia-Telangiectasia mutated* (ATM) gene, a serine/threonine kinase, which is activated in response to DNA double strand breaks^44^. ATM naturally exists as a resting inactive dimer which is auto-phosphorylated into an active monomeric form that interacts with the MRE11/RAD50/NBS1 (MRN) complex at the site of strand breaks, co-ordinating DNA repair^45,46^. ATM also phosphorylates multiple downstream targets (notably including, p53, γ-H2AX, CHK1 and CHK2) that are also involved in apoptosis, cell cycle regulation and DNA repair^47–49^. The mechanism during oxidative stress is different to the DDR response, in that ATM forms a disulphide linked stable dimer activated independently of MRN and DNA damage^50,51^. Despite being a predominantly nuclear protein, several studies have indicated that during elevated ROS that ATM may translocate through the nuclear membrane to interact with cytosolic proteins localised in the peroxisome, mitochondria and microsome^52–54^. The importance of ATM in the cellular response to oxidative stress can be seen by investigating its role in the autosomal recessive disorder *ataxia telangiectasia* (A-T), after which ATM is named^55^. A-T patients lack fully functioning ATM and are predisposed to a range of malignancies, radiation sensitivity, immunological and neurological disorders^56^ caused by abnormalities in the cells response to DNA repair and elevated ROS levels particularly in neurons^57^. Studies in ATM-deficient mouse models have also shown similar abnormalities^58^. Interestingly ATM deficient patients can sometimes present with early aging phenotypes including canities or with the pigment disorder vitiligo^56^ and mice lacking ATM can also display coat and tail pigmentation abnormalities particularly after genotoxic insult^59^. Inomata et al*.* investigated the effects of ATM knockout on murine hair follicles and described niche depletion of melanocyte stem cells from the bulge region of the hair follicle in response to genotoxic stress^10^. ATM deficiency caused sensitization and early differentiation of melanoblasts leading to depletion from the stem cell niche which caused depigmentation of the follicle in subsequent hair cycles. Arck et al*.,* also proposed the ‘free radical theory of greying’ which described increased apoptosis, reduced oxidative stress protection and increased mitochondrial DNA damage in hair follicles leading to depletion of melanocytes and greying hair^60^.

Despite studies investigating stress, ROS or pigmentation markers in both pigmented and canities affected hair follicles, information on the collective status, expression and distribution of selected cell cycle, DNA damage, apoptosis and ROS metabolising anti-oxidant markers in aging human scalp follicles is limited. In this study we have analysed the protein expression of over 20 markers of cell cycle, DNA damage, apoptosis, senescence and oxidative stress in hair bulb melanocytes and in the wider growth scalp hair follicle in normal healthy individuals across six decades. Here we show distinct nuclear expression of ATM within (surviving) melanocytes in canities-affected hair bulbs of the hair follicle, the frequency of which correlated with hair follicle pigmentary status. We found infrequent expression of other markers of senescence, DNA damage/repair and oxidative stress specifically within the bulbar melanocytes. A prominent feature of this study was the strong expression of markers for apoptosis/(terminal) differentiation and ROS activity for the anagen hair follicle’s inner root sheath (IRS). In vitro analysis of cultured human hair follicle melanocytes showed that ATM is a marker of oxidative stress in these cells. ATM appears to play a role in protecting human hair follicle melanocytes from oxidative stress/damage, and maintenance of its expression within the follicle microenvironment along with other redox markers may be important for hair bulb melanocyte survival in canities-prone scalp hair follicles.

## Methods

### Tissue collection and approval

Research was carried out according to local University of Bradford (UoB) good research practice guidelines. Healthy normal human haired scalp (age range 22–70 years) was sourced from elective (cosmetic) plastic surgery. Tissue was obtained with regulated informed consent from all individuals by the UoB Ethical Tissue Bank (an ethically approved human research tissue bank, licensed by the Human Tissue Authority (HTA), Licence number: 12191) with approval from the National Research Ethics Service (NRES) Committee Yorkshire & The Humber—Leeds East (approval number 17/YH/0086). Tissue was placed in transport media (DMEM containing L-glutamine, 10% FCS, 5 × Pen/Strep, 3 × antifungal) immediately after removal. Samples were cleaned in wash solution (phosphate buffered saline (PBS) containing 5 × Pen/Strep with antimycotic/antifungal) and hair was shaved off close to the epidermis.

### Double immunohistochemistry of scalp tissue

This assay was conducted as described previously and adapted to the antibodies used^61^. Briefly, ten micron sections were air-dried onto slides for at least 1 h prior to fixing in ice-cold acetone for 10 min at − 20 °C. Non-specific antibody binding was reduced by incubating in 10% donkey serum (Sigma-Aldrich, UK) diluted in PBS for at least 30 min, followed by simultaneous incubation in primary antibodies (a melanocyte marker with a target antibody; see Supplementary table 1) diluted in PBS containing 1% donkey serum overnight at 4 °C. After washing sessions in PBS for 3 × 10 min, these were then incubated in donkey Alexa-488 and 594 conjugated secondary antibodies (1:100 dilutions, Thermofisher, UK) for 1 h at RT. After washing the sections were mounted for confocal microscopy under sealed coverslips in fluorescent mounting medium containing DAPI nuclear stain (VectorLabs, UK). Images were collected using the 365 nm (DAPI), 488 nm (Alexa-488) and 543 nm (Alexa-594) channels on a Zeiss LSM confocal microscope by sequential line scanning. Images were processed using the LSM confocal image browser software (Zeiss, UK) and ImageJ (freeware).

### Isolation of human scalp hair follicle melanocytes

Isolation and purification of hair follicle melanocytes (HFMs) from scalp hairs was carried out as originally previously described in Tobin, et al*.*^62^ with modifications^63^. Cell lineage identity was confirmed by GP100 and TRP-1 expression. Melanocytes were maintained in full 2:1 melanocyte growth media (2 parts minimal essential medium (MEM) supplemented with 1% foetal bovine serum (FBS), 1% nonessential amino acids, 100 µg/ml Primocin, 2 mM Glutamax, 5 ng/ml basic fibroblast growth factor, and 5 ng/ml endothelin-1 (Sigma, Dorset, UK) mixed with 1 part keratinocyte serum-free medium (K-SFM) supplemented with 25 µg/ml bovine pituitary extract (BPE), 0.2 ng/ml rEGF, 100 µg/ml Primocin (antibiotic), and 2 mM Glutamax in humidified incubators at 37 °C with 5% CO_2_ unless stated otherwise.

### Treatment of hair follicle melanocytes in culture

Prior to treatment, primary HFMs were incubated for 24 h in ‘starved’ 2:1 melanocyte media (2:1 melanocyte media minus 1% FBS and BPE). All treatments were carried out in starved 2:1 melanocyte media for the duration of experiments. Pro-oxidants: fresh hydrogen peroxide (Sigma, UK) solution was prepared as a 10 mM stock solution immediately before use then diluted to the required concentration. Menadione (Sigma, UK) was dissolved in DMSO to 100 mM then diluted to 100 µM in media immediately prior to use. Antioxidants: Vitamin E and Quercetin (VitE/Q; Sigma, UK) were dissolved in water (1 mM) and DMSO (10 mM) respectively before combining to a final concentration of 10 µM (Vitamin E) and 1 µM (Quercetin) in media. Cells were pre-incubated with VitE/Q for 1 h prior to ROS induction. The ATM specific kinase inhibitor KU60019 (Tocris, UK) was dissolved in DMSO to 100 mM then diluted to 5 µM directly into media. Cells were pre-incubated in KU60019 for 1 h prior to ROS induction.

### Quantification of ATM expression

Cultured hair follicle melanocytes were seeded into 8-well chamber slides (~ 10,000 cells/well) and left to attach for at least 24 h, fixed in ice-cold methanol for 10 min, and incubated with blocking buffer of 10% donkey serum (Sigma-Aldrich, UK) for at least 30 min. After washing, this was followed by incubation in anti-ATM primary antibody [Y170] (Abcam, Cat# ab32420) diluted 1:100 overnight at 4 °C and then incubated in donkey Alexa-488 conjugated secondary antibodies (1:100 dilution, Invitrogen Molecular Probes, UK) for 1 h at RT. The slides were mounted under medium containing DAPI (VectorLabs, UK) for confocal microscopy. Images were collected using the 365 nm (DAPI) and 488 nm (Alexa-488) channels on a Zeiss LSM confocal microscope by sequential line scanning. Images were processed using the LSM confocal image browser software (Zeiss, UK) and ImageJ (freeware).

### Western blot analysis

Primary HFMs were seeded into 6-well plates (~ 500,000 per well) and left to attach overnight. Prior to treatment, cells were incubated in starved media for 24 h (2:1 melanocyte media minus FCS and BPE). After treatment, media was removed and cells rinsed in PBS before lysis with 200 μl ice-cold RIPA buffer (Sigma, UK) containing protease inhibitors (Complete EDTA protease inhibitor tablets; Roche, UK) and phosphatase inhibitors (phosphatase inhibitor tablets; Pierce, UK). Cell lysates were sonicated briefly for 15 s then protein concentration measured using the Microplate BCA. Protein Assay Determination Kit—Reducing Agent Compatible (Pierce, UK). Lysates were diluted in 2 × Laemelli SDS sample buffer containing 50 mM DTT to equal concentration then ~ 10 μg of protein electrophoresed through NuPage 3–8% Tris–Acetate gels (Thermofisher Scientific) alongside Kaleidoscope (Bio-Rad, UK) and HiMark (Invitrogen, UK) protein standards. Protein was transferred to PVDF membrane (Mini Trans-blot Turbo Transfer Pack) using the Trans-blot Turbo module at 2.5 A constant (~ 25 V) for 30 min. PVDF membranes were cut to size and blocked in 5% (w/v) non-fat milk powder dissolved in TBS-T (TBS-Tween 20 (0.01% v/v)) for at least 1 h at room temperature (RT). Membranes were incubated in either Abcam Cat# ab32420 Rabbit anti-ATM [Y170] antibody diluted 1:4000 in 5% milk/TBS-T overnight at 4 °C, then rinsed in TBS-T. Membranes were then incubated for 30 min at RT in HRP-conjugated secondary antibody (Santa Cruz Biotechnology, Inc, USA) diluted in 5% milk or BSA in TBS-T. Separated membranes containing WesternC marker were incubated in Streptavidin HRP-conjugated label (1:20,000) and secondary antibody only. Excess secondary antibody and Streptavidin-HRP was removed by washing in 4 changes of TBS-T. Cut membranes were pieced back together and incubated in Clarity ECL detection reagent (Bio-Rad, UK) for 5 min then imaged using the ChemidocMP imaging system (Bio-rad, UK). Protein bands were quantified by densitometry using Image Lab v4.1 software (Bio-Rad, UK). For Catalase and GP-1 Western blotting samples were prepared as described earlier, however proteins were electrophoresed through Bio-Rad Any kD mini-TGX PAGE gels alongside Kaleidoscope and Western C markers (Bio-Rad, UK), then transferred to PVDF using a Turbo Transfer blotter (2.5 mA, 25 V constant for 10 min). Membranes were blocked as earlier in 5% Milk/TBS-T then probed overnight with mouse anti-Catalase primary antibody (Sigma #C0979, diluted 1:1000 in 5% milk/TBS-T) at 4 °C then rinsed in TBS-T buffer. Membranes were then incubated for 30 min at RT in HRP-conjugated secondary antibody (Santa Cruz, Biotechnology, Inc, USA) diluted in 5% milk in TBS-T and imaged as described previously. Where necessary, membranes were stripped using Restore reagent (Thermofisher, UK) then reprobed for the housekeeping gene GAPDH (1:2000 Cat# MCA4740, Bio-Rad, UK) as a loading control.

### Cell viability test

Primary HFMs were seeded into 96-well plates at a density of 1 × 10^4^ cells/well (n = 12) in full 2:1 Melanocyte media. Cells were incubated at 37 °C with 5% CO_2_ overnight to attach to plates, rinsed in PBS, then placed in starved media overnight before treatment. Treatments were prepared immediately prior to addition with a final dilution into starved media then added to cells. DMSO vehicle (basal) controls (0.00005% v/v) were also used in parallel. Following incubation, cells were rinsed in PBS then incubated with WST-1 reagent (diluted 1:10 into fresh starved media (Roche, UK)) and incubated at 37 °C for 4 h in a cell culture incubator according to manufacturer’s instructions. Plates were scanned for absorbance at A_450nm_ on a Tecan M200 plate reader using a reference wavelength of A_690nm_. Results were analysed using Magellan 6.0 software (Tecan, Germany). Mean absorbance relating to cell viability was calculated then plotted on a bar chart (Graphpad Prism v6.0).

### Statistics

Data were expressed as mean ± SEM. Error bars also show SEM. Differences between data were evaluated by Student’s t test or ANOVA where stated after testing the data normality using Graphpad Prism v6.0.

## Results

Human scalp from multiple normal healthy donors was assessed for the expression of canities-relevant modulators of melanocyte phenotype using a panel of 23 different antibodies by immunofluorescence. Antibodies were directed against proteins implicated in key pathways such as apoptosis, cell cycle regulation, senescence, DNA repair/damage and oxidative stress. Double-staining was performed using a melanocyte-specific marker to investigate colocalization in bulbar HFM. Brightfield imaging was used in parallel to monitor the pigmentation status (melanin level) of each stained hair bulb.

### Expression of melanocyte specific markers in greying human hair follicles

**Figure 1:**
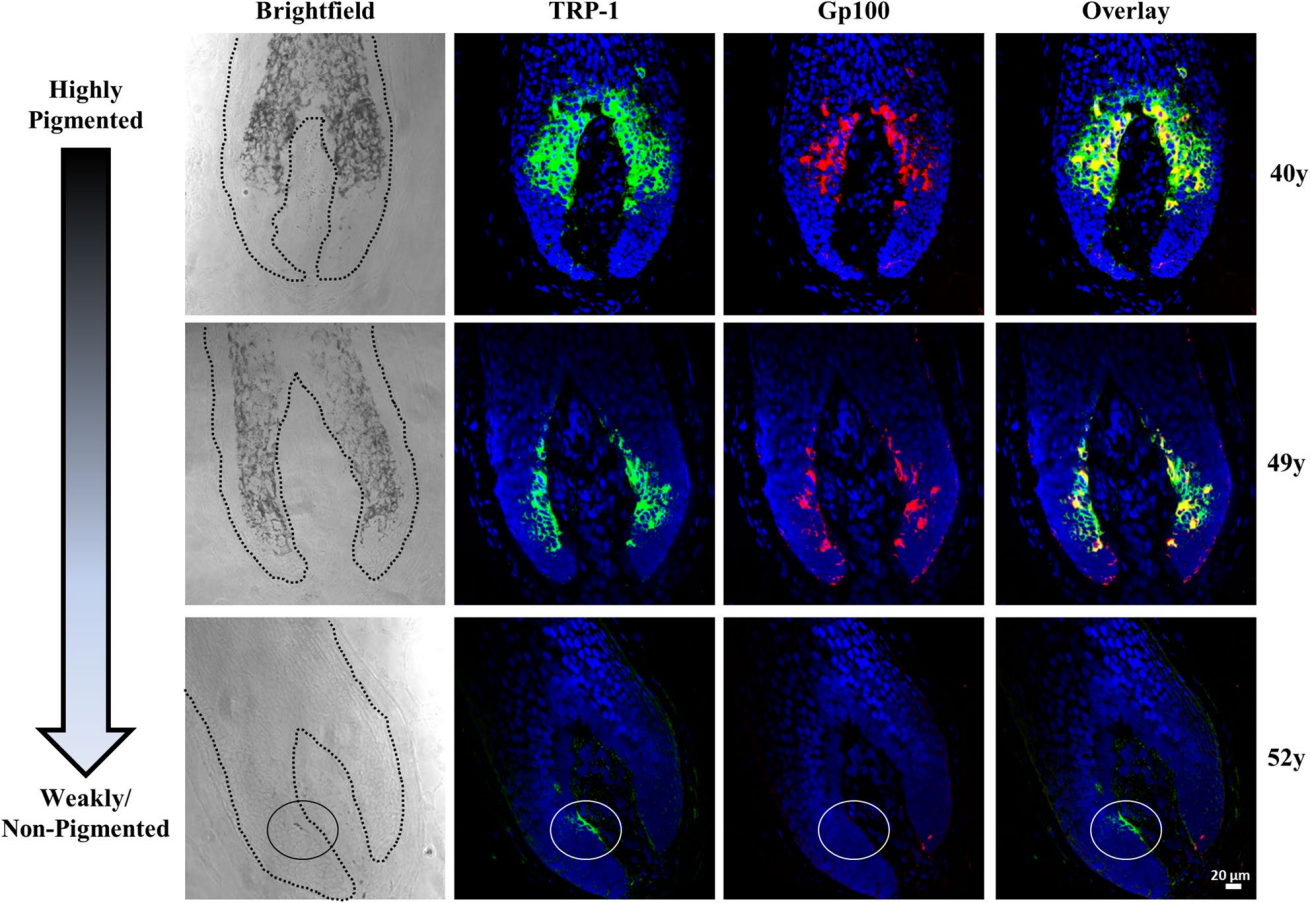
Expression of melanocyte markers GP100 and TRP-1 is reduced in proportion to canities extent. Double IF of scalp tissue with TRP-1 (green channel) & GP100 (red channel) shows strong staining of both markers with a gradual decrease in melanocyte labelling from highly pigmented to weakly non-pigmented grey hair follicles. Melanocyte marker expression was proportional to the melanin content observed in brightfield images (left panels; hair follicle outline is indicated with black dashed line). Circles in lower panels show the last remaining TRP-1 positive/GP100 negative staining of isolated melanocyte(s) localised to a small region of the inner hair bulb with near complete (fully grey) canities (which is still pigmented in the brightfield image). DAPI nuclear stain is shown in blue channel. Scale bar = 20 µm. Donor age is indicated on the right.

The correlation between the pigmentation status and the melanocyte-specific markers (GP100, Tyrosinase related protein-1 (TRP-1) and Tyrosinase (TYR) was evaluated in human scalp hair bulbs. The first observation was that scalp tissues from donors aged from 22 to 70 years old, contained variably pigmented follicles. Multiple donors had fully pigmented follicles co-located with intermediary to weakly/non- pigmented hair follicles (data not shown). Comparison of melanocytes staining with melanin content of hair bulbs showed the expected gradual decrease in melanocyte labelling from fully pigmented to weakly/non-pigmented hair bulbs (Fig. 1 and supplementary figure 1). The intensity and the distribution level of the staining of TRP-1 and GP100 was higher in the hair follicle melanocytes of fully pigmented follicles, sporadic in melanocytes with reduced pigmentation and lacking in weakly pigmented/white hair follicles (Fig. 1). The intensity of staining of TYR was also correlated to the pigmentation status of the hair bulb. However, the protein exhibited lower levels of expression and distribution in the hair bulb melanocytes compared to those of TRP-1 and GP100. For instance, TYR staining was markedly reduced in the melanocytes of hair follicles with mild or moderate canities, despite strong TRP-1 expression in the same hair follicle (see supplementary figure 1). The lack of full overlapping between the three-melanocyte lineage-specific markers suggests differential labelling of melanocyte populations. The melanocyte-specific marker TRP-2 was also investigated. However, the hair bulb of anagen hair follicles lacked TRP-2 expression as previously reported^64^.

### ATM staining correlates with pigmentary status in hair follicles

**Figure 2:**
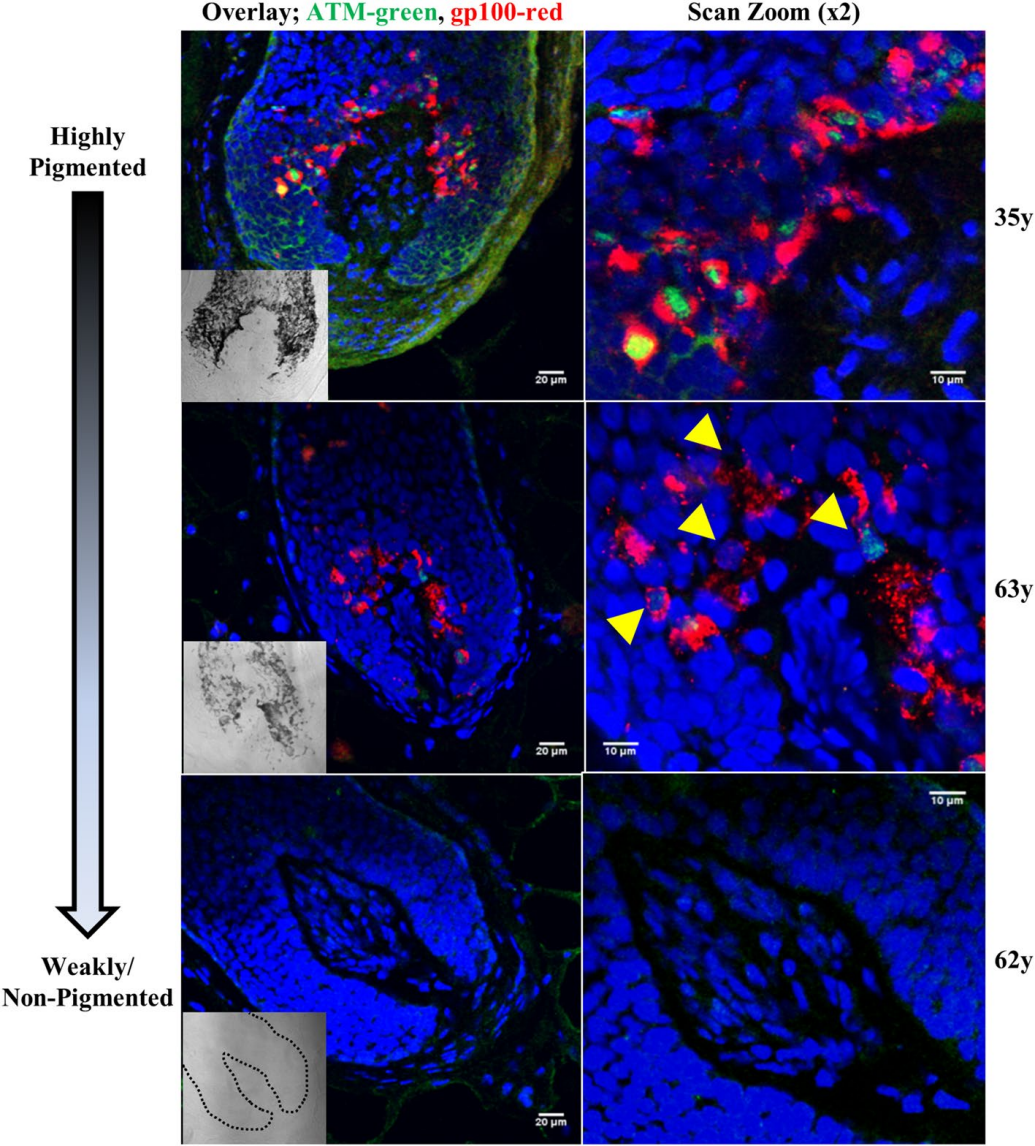
ATM is expressed within hair follicle bulbar melanocytes and correlates with pigmentation status. Overlays of ATM (green channel) and melanocyte marker GP100 (red channel) in adult human scalp showing strong and exclusive ATM expression in melanocytes within a pigmented follicle (top, 35-year old), less frequent ATM expression in a hair follicle with reduced pigmentation or mild canities (middle, 63-year old), and absent ATM expression in a grey (full canities) hair follicle (lower; 62-year old). Right panels show increased magnification (× 2) of the hair bulb melanocytes present in the central column. Yellow arrows indicate melanocytes with reduced or absent ATM expression in canities-affected scalp follicles. DAPI nuclear stain is shown in blue channel. Scale bar is shown on each image. Brightfield images of follicle pigmentation are shown in the inset image (follicle outline is indicated with black dashed line in 62-year old with full canities). Donor age is indicated on the right.

**Figure 3:**
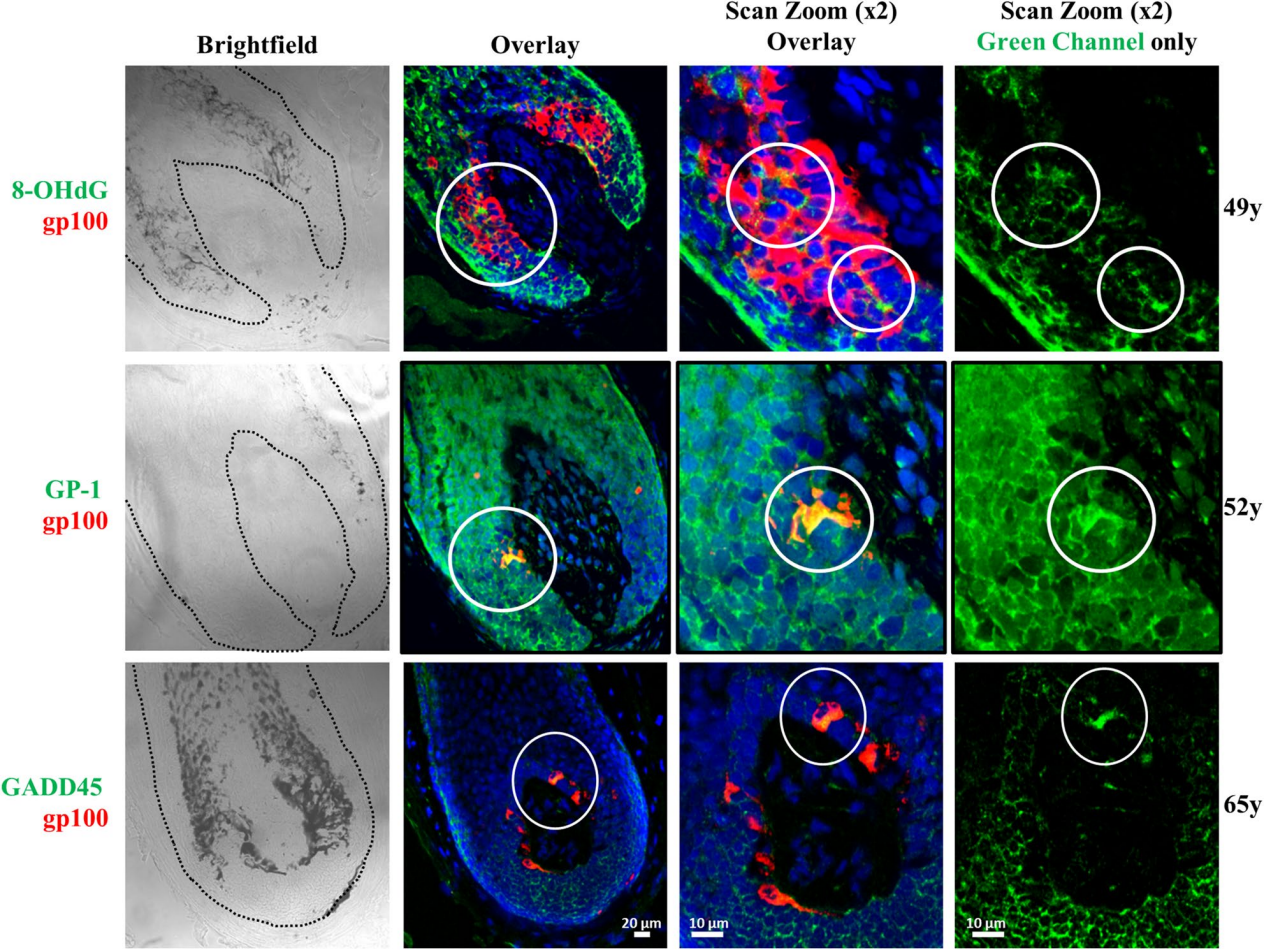
Colocalisation of 8-OHdG, GP-1 and GADD45 in occasional hair bulb melanocytes. Double IF staining in scalp tissue from 3 donors showing 8-OHdG (top; 49-year old), GP-1 (middle; 52-year old) or GADD45 (lower; 65-year old) expression in green channel colocalised with expression of the melanocyte marker GP100 (red channel). DAPI nuclear stain is shown in blue channel. Brightfield images on the left panels show the pigmentation status of each hair follicle (the follicle outline is indicated with a black dashed line). Right middle panels show increased magnification overlay (× 2) of the hair bulb regions. Far right panels show green channel staining only. Circles indicate increased protein expression in isolated melanocytes. Scale bar = 20 µm for overlay images and 10 µm for scan zoom (× 2) images.

A major finding of the immunofluorescence study on scalp tissues was that in addition to the melanocyte specific markers, another protein appears to be strongly related to the hair bulb melanocytes and their pigmentary status. Expression of the DNA-repair coordinator and oxidative stress sensor ATM was exclusively restricted to hair follicle melanocytes (i.e. was not expressed in follicular keratinocytes and fibroblasts) with a pattern that correlated with pigmentary status of the hair follicle within the hair bulb (Fig. 2). Heavily pigmented hair follicles showed strong nuclear ATM colocalisation within bulbar melanocyte nuclei. In hair follicles with reduced overall pigmentation, hair bulb melanocytes displayed variably reduced expression or absent ATM expression. ATM expression was completely absent in white hair follicles without pigmented cells (*i.e*. full canities). It was also noted that frequent strong ATM nuclear staining was colocalised within the nuclei of GP100 positive melanocytes and occasional basal keratinocytes in the UVR exposed epidermis of multiple scalp samples. Nuclear ATM staining was not detected within dermal fibroblasts (supplementary figure 2), and although some cytoplasmic expression was visible. Among the biomarkers implicated in cell cycle, DNA damage/repair, apoptosis, senescence and oxidative stress, only ATM was consistently expressed in hair follicle melanocytes in situ and correlated to the pigmentary status of the hair bulb. By contrast, only occasional colocalisation with isolated bulbar melanocytes was detected for GP-1, 8-OHdG and GADD45; however, their expression pattern or level did not correlate strongly with overall pigmentary status or with age of donor (Fig. 3). All other markers tested did not appear to be expressed to any significant extent with melanocytes.

### Novel expression of multiple markers for ROS and apoptosis in differentiating inner root sheath (IRS) of anagen scalp hair follicles

A striking feature of this study was the intense but highly nuanced expression of markers for ROS and apoptosis/(terminal) differentiation in the IRS (supplementary figures 3a–f), including Caspase-2 (CASP-2) and Caspase-9 (CASP-9), (supplementary figures 3a and 3b respectively), the DNA damage marker GADD45 (supplementary figure 3c), p21 (supplementary figure 3d), p57 and phospho-p57 (supplementary figure 3e), GR-1 (supplementary figure 3f.) and HO-1 (supplementary figures 3g and 3h). A component of the anti-oxidant glutathione metabolic pathway GR-1 was expressed predominantly in mature Henle’s layer of the hair follicle IRS (supplementary figure 3f.). By contrast, the stress sensor GADD45 was detected within only early differentiating IRS cells (supplementary figure 3c), while the ROS-protective protein heme oxygenase-1 (HO-1) was intensely expressed in a small subset of IRS cells located usually in the upper follicle, at the level of the sebaceous gland, where IRS cornification is advanced to complete (supplementary figures 3g and 3h). Variable expression of several markers was also detected elsewhere in the anagen HF as follows: dermal papilla (BCL-2, GP-1), hair follicle outer root sheath ORS (p53, p57 and phospho-p57), hair follicle pre-cortex (CASP-2, SOD-1, GADD45), hair bulb keratinocytes (GP-1, GADD45, 8-OHdG) and dermal sheath (CASP-2). Low levels of p38 α/β stress kinase were detected in early canities in two donors although it was unclear if it was located within peripheral hair follicle melanocytes or in DP cells. We did not detect any CASP-3, phospho-p53, p27/phospho-p27 and phospho-p38 expression in these samples.

### ATM protein levels increase in hair follicle melanocytes in vitro in response to elevated oxidative stress

**Figure 4:**
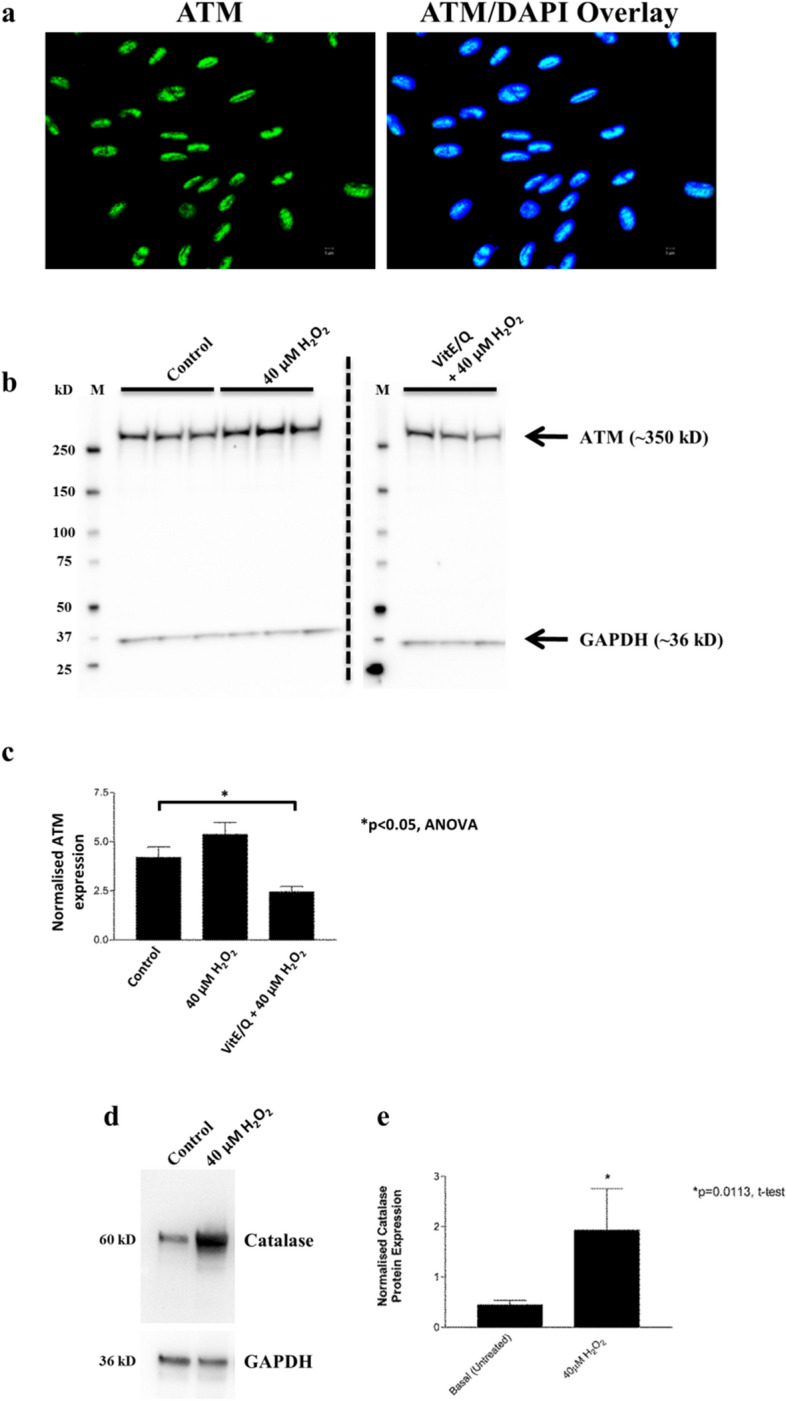
(**a**) ATM is expressed in HFM’s in vitro. Immunocytochemistry staining showing the nuclear expression of ATM protein (green channel; left image) in cultured primary hair follicle melanocytes (from male scalp, 20-year old). DAPI nuclear stain (blue channel) is co-localised with nuclear ATM staining on the right-hand side image. Scale bars are shown on each image. (**b**) and (**c**) ATM expression is increased in HFM after exposure to oxidative stress and can be reduced by antioxidants. HFM were treated with 40 µM H_2_O_2_ for 1 h in parallel with untreated control cells and cells pre-treated for 1 h with the antioxidants VitE/Q prior to H_2_O_2_ addition. Levels of ATM protein expression (~ 350 kD) were assessed by Western blot (**b**). Antioxidant-treated samples were loaded on a second gel and processed in parallel with other samples under identical conditions. GAPDH (~ 36 kD) was used as a loading control. Marker is shown in the first lane of each gel (M). An increase in ATM protein expression could be seen in H_2_O_2_ treated samples compared to control untreated cells (ns). Levels of ATM could be significantly decreased by preincubation with VitE/Q for 1 h before H_2_O_2_ treatment (*p < 0.05 versus control, ANOVA; n = 3). Graph in c shows quantification of mean ATM expression (normalised to GAPDH in relative densitometry units) from the Western blot. (**d**) and (**e**) Catalase expression is increased in HFMs after exposure to hydrogen peroxide. Representative Western blot showing induction of catalase protein expression (~ 60 kD) in HFM’s after treatment for 1 h with 40 µM hydrogen peroxide. GAPDH (36kD) was used as a loading control. Graph in e shows quantification of mean catalase expression (normalised to GAPDH in relative densitometry units) from Western blots after 40 µM H_2_O_2_ treatment (n = 3). Unpaired t-test (*p = 0.0113) compared 40 µM H_2_O_2_ treated cells versus controls.

In vitro analysis of primary hair follicle melanocytes (HFMs) isolated from scalp tissue showed strong nuclear expression of ATM (Fig. 4a). To investigate if ATM protein levels change in these melanocytes during elevated oxidative stress, these cells were treated with 40 µM H_2_O_2_ for 1 h to induce ROS. Protein levels of total ATM and the ROS-activated S1981-phosphorylated ATM were investigated using Western blot analysis. Results showed increased total ATM levels in HFMs after H_2_O_2_ treatment, and this increase was blocked by preincubation of HFMs with antioxidants VitE/Q for 1 h (*p < 0.05 VitE/Q pretreatment versus untreated control; Fig. 4b,c). However, expression of ROS-activated phosphor-ATM S1981 could not be detected despite elevated levels of catalase in these cells in response to H_2_O_2_ treatment (Fig. 4d,e and supplementary figure 4).

### Inhibition of ATM kinase activity in HFM in vitro reduces cell viability

**Figure 5:**
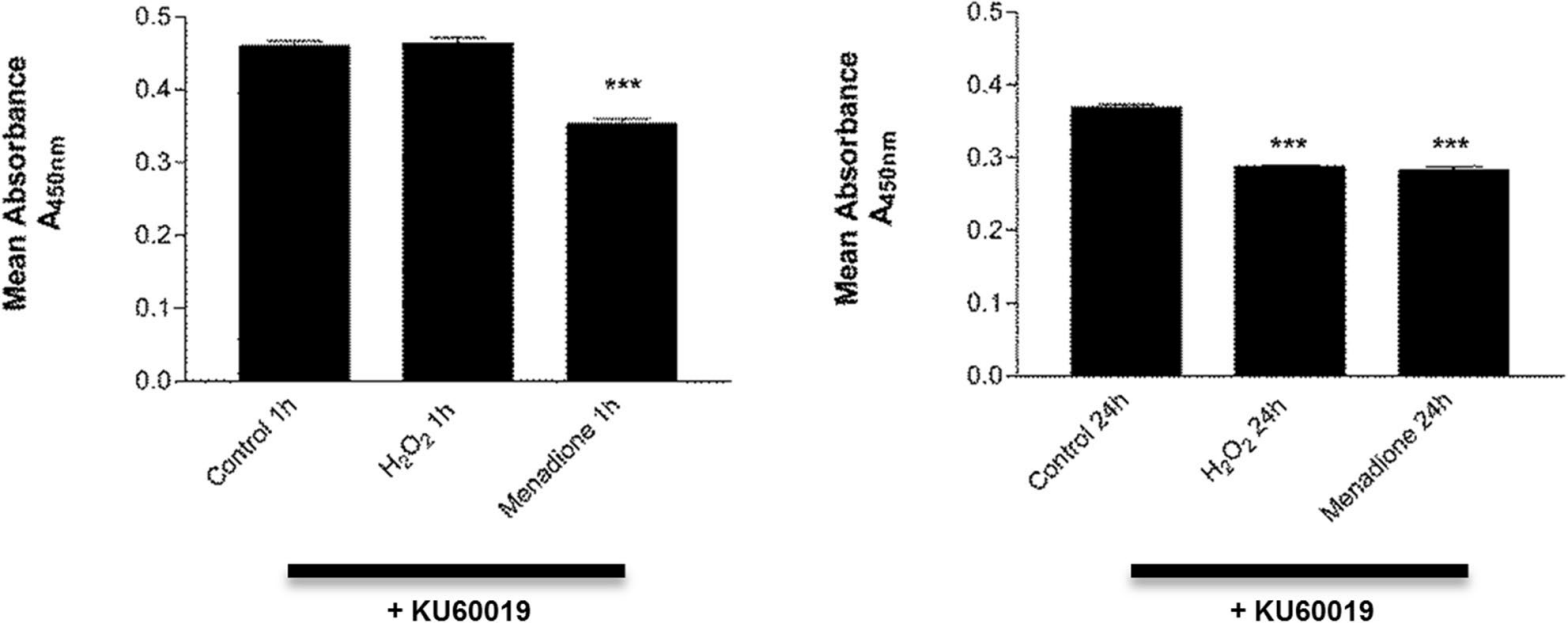
ATM kinase activity is necessary for cell survival and viability during sustained elevated oxidative stress. Melanocytes were pre-treated with the ATM specific kinase inhibitor KU60019 (5 µM) for 1 h then incubated in either 250 µM H_2_O_2_ or 100 µM Menadione (a known potent inducer of oxidative stress) for short (1 h) or long (24 h) time periods and cell viability assessed using the WST-1 assay (n = 8) by measuring absorbance (A_450nm_) of mitochondrial generated formazan, the amount of which is directly proportional to the number of living cells. Left graph shows the results of 1 h treatments with each ROS inducer. Menadione treatments showed a significant reduction in cell viability (***p < 0.001, ANOVA) versus vehicle controls. Right graph shows the results of 24 h treatments with each ROS inducer. Both Menadione and H_2_O_2_ treatment significantly reduced cell viability (***p < 0.001, ANOVA) versus vehicle controls.

To investigate if ATM activity influenced melanocyte survival under sustained elevated ROS conditions in culture we pre-treated cells with the ATM-specific kinase inhibitor KU60019 (which abrogates the kinase function of the protein and prevents downstream phosphorylation of ATM targets^65^) and incubated these cells with an increased concentration of 250 µM H_2_O_2_ (to negate the effects of rapid H_2_O_2_ deactivation in vitro ensuring sustained elevated ROS during longer treatment times) and 100 µM menadione (a naphthoquinone that can generate ROS through redox cycling^66^) to raise ROS levels, for short (1 h) and long (24 h) durations then measured cell viability. As seen in Fig. 5, results showed that in the absence of ATM kinase activity, sustained H_2_O_2_ treatment for 24 h significantly reduced cell viability compared to controls (***p < 0.001 ANOVA). Treatment with the potent ROS inducer menadione^67,68^ also significantly reduced cell viability at both 1 h and 24 h time points compared to untreated controls (***p < 0.001 ANOVA). Treatment of HFM with only 5 µM KU60019 was found to reduce total mean ATM protein levels compared to untreated cells (supplementary figure 5).

## Discussion

Despite several studies into the biology and genetics of hair greying or canities, the mechanism by which human scalp hairs lose pigmentation remains poorly understood, due in part to the complexity of interaction between different cell types, complex protein interactions, differential gene expression during the hair growth cycle, and variable conditions that occur within the hair follicle microenvironment^1,7–10,13,41,60,69–74^. Here we have used a specific panel of antibodies chosen on the basis of their involvement in DNA repair, oxidative stress, cell cycle dynamics, senescence and apoptosis to investigate potential markers of canities within hair bulb melanocytes in the scalp of multiple normal healthy donors aged from 22 to 70 years old. One of the major findings of this study was that of all the biomarkers tested, only ATM was found to be exclusively expressed within hair bulb melanocytes and so correlated with hair follicle pigmentation status. ATM nuclear expression markedly decreased in the melanocytes of hair follicles with reduced pigmentation. ATM was also absent in some hair bulbs that contained few remaining GP100 positive melanocytes.

ATM is a PI3K-kinase and one of the major components of the DNA damage response (DDR) pathway^46^ but can also be activated during oxidative stress^50,51^. It exists as an active dimer^45^ and targets phosphorylation of downstream targets such as CHK2, γH2AX and p53^44,47–49^. Investigations on primary hair follicle melanocytes (HFM) in vitro showed that total levels of ATM were upregulated after exposure to hydrogen peroxide that increased oxidative stress. Nishimura and colleagues showed that loss of ATM in melanoblast stem cells within the hair follicle bulge region in mice could sensitize the stem cells to genotoxic stress, leading to early differentiation, depletion from the niche and progressive hair greying over subsequent cell cycles due to eventual depletion of mature melanocytes from the hair bulb^3,10^. Oxidative stress is associated with the auto-phosphorylation of ATM at S1981^51^, but this was not detected by Western blot analysis in the current study. Hydrogen peroxide is known to be a short-lived molecule in cell cultures that is degraded within 30 min. However during that time it can be a potent activator of cellular anti-oxidant response systems, and if high enough can even induce DNA damage and cause cell death^75^. It has been speculated that when peroxide activates ATM in vitro little autophosphorylation takes place and it may be partially dependent on complexing with the MRN complex recruited to DNA repair^51,76^. This may explain why we could not detect ROS activated S1981 phosphorylation of ATM, particularly if the H_2_O_2_ concentrations were not high enough to induce DNA damage in HFMs. To determine if levels of ROS were increased during treatment’s we measured catalase and GP-1 proteins levels by Western blot and both were markedly increased in HFM’s after addition of H_2_O_2_ indicating that the ROS stress response within cells was active. If auto phosphorylation of ATM S1981 is absent and the antioxidant response is functioning in HFM then does ATM play a role in melanocyte survival during ROS induced stress? To investigate this we used the ATM specific kinase inhibitor KU60019^65^ to inhibit the downstream phosphorylation activity of ATM with both H_2_O_2_ and menadione. The naphthoquinone menadione is metabolized by several enzymes through one electron reduction resulting in highly reactive unstable semiquinones^77^. These enter redox cycles to reform quinones but produce high amounts of free radical oxygen that are subsequently deactivated by dismutase’s, producing elevated H_2_O_2_^67,68,77^. We detected increased sensitivity to ROS and decreased cell viability in both cases, especially after a sustained increase in oxidative stress over 24 h. This indicates that although ATM may not be activated through traditional ROS associated S1981 phosphorylation in HFM, functional ATM is still important to bulbar melanocyte survival during elevated oxidative stress. The exact mechanism by which ATM is activated by and which downstream proteins are targeted needs further investigation. Casual observation during treatments showed that HFM appeared to exhibit mild cytotoxicity effects to external H_2_O_2_ around the 250 uM concentration and levels above this would produce mass cell death/apoptosis in cultures within 24 h of treatment. We postulate that functional melanocytes may have multiple intrinsic robust mechanisms to process H_2_O_2_ induced ROS due to the natural generation of H_2_O_2_ during melanogenesis itself, although further work is needed to investigate this. KU60019 has been well documented as a very potent ATM kinase inhibitor, used in multiple ATM publications at similar concentrations as our study. Thus, we would expect ATM activity to be completely suppressed^78–86^. Treatment of HFM with the KU60019 kinase inhibitor alone reduced total mean ATM protein levels compared to untreated cells. This would be expected as other studies have documented that ATM phosphorylation can increase total ATM expression through a feedback mechanism^87–89^. However, we cannot completely rule out secondary effects on closely related cell signalling pathways such as ATR/Chk1 mediated DNA repair which can in part be influenced by reductions in ATM kinase activity^90,91^.

In our study we only detected co-localisation and increased staining of three other proteins (GP-1, GADD45 and 8-OhDG) within bulbar melanocytes. Other protective mechanisms may cooperate with ATM expression/activity to protect bulbar melanocytes from oxidative stress-induced terminal differentiation similar to melanocyte stem cells within the murine hair follicle niche^10^. Kauser et al., showed that reduced expression of catalase in HFM cultured from elderly donors compared to HFM isolated from young donors, indicating that catalase expression/activity is an important effector in the response of melanocytes to aging-associated stimuli over time^92^. Other studies have shown the importance of the NRF2/HO-1 proteins in directing the ARE (anti-oxidant response elements) pathway in melanocytes in response to H_2_O_2_ induced stress^41,42^. Coincidently, ATM is thought to regulate selective mitochondrial functions through NRF1 (a similar protein of the NRF2 family) in response to ROS^93^. It has been shown that melanocytes can produce peroxide as a consequence of melanin synthesis^25–28^. Hair bulb melanocytes already have robust mechanisms in place using the cells own intrinsic anti-oxidant enzymes to reduce elevated ROS before critical levels that damage DNA occur. We would speculate that ATM is part of a larger cohort of proteins that are tightly regulated to cope with elevated ROS levels and that together they contribute to protecting melanocytes from oxidative stress and ensure their survival within the hair bulb. Changes in expression levels of these marker proteins could imbalance ROS levels, leading to a gradual reduction in melanocyte protection and viability over time leading to hair greying. As loss of ATM correlates with decreased pigmentation in hair follicle melanocytes it is difficult to determine if loss of melanin contributes to a reduction in ATM expression or drives the process of ATM loss more rapidly. Melanin production is known to decrease and become less efficient with aging in melanocytes, with fewer melanosomes produced and poor quality/quantity of melanin^8,71^. We also specifically analysed scalp tissue from skin types II-III in this study and so could not detect differences between eumelanotic and phaeomelanotic phenotypes. Further work is therefore needed to investigate this.

Elsewhere in the hair follicle, several other cell cycle, DDR, apoptosis and ROS metabolising anti-oxidant markers appear to have very specific expression in specialised cell layers of the hair follicle, particularly those cells undergoing differentiation in the anagen-specific IRS during hair fibre formation. Different compartments or cell layers of the hair follicle may have their own individual ‘microenvironment’, with varied requirements to protect cells from ROS caused by normal cell activity such as differentiation. Lemasters et al., showed that hair follicles have specific compartments of mitochondrial gradients and oxidative metabolism, which leads to increased ROS during the development of the hair shaft that they termed a ‘ring of fire’^94^. Another study showed that mice with a keratinocyte-specific deficiency in mitochondrial transcription factor A (TFAM), a gene required for the transcription of mitochondrial genes, have abnormalities in hair follicle growth, and that mitochondria-generated ROS was in fact necessary for the mediation of cell differentiation^95^. Indeed, a high proportion of the markers tested in our study localised to where cells differentiate to establish the IRS, and it appears their presence is necessary to prevent damage or to stabilise ROS in cells during the differentiation process. Further studies are needed to determine if regulated expression of p21, CASP-2, CASP-9, SOD-1 and HO-1, components of the anti-oxidant glutathione metabolic pathway GP-1 and GR-1, and the DNA damage marker GADD45 are necessary to complete differentiation in specialised keratinocyte layers.

In summary we have shown that ATM protein expression correlates with pigmentation status in greying hair follicles and contributes to sustained melanocyte viability in vitro during elevated ROS levels. We have also showed that other markers of DNA damage/cell cycle (p21, p53, p57 and phospho-p57, GADD45, 8-OHdG), apoptotic (BCL-2, CASP-2, CASP-9) and anti-oxidant pathways (GP-1, SOD-1, HO-1, p38 stress kinase), that are not specifically associated with hair follicle pigmentation, have distinct expression patterns and colocalise to parts of the hair follicle such as the IRS, ORS, DP and cortex. These results highlight the multicellular complexity of the hair follicle to cope with oxidative stress and indicate novel previously unreported roles for several proteins implicated in cell cycle/senescence and oxidative stress in IRS differentiation pathways and hair follicle growth. Further work is needed to establish how ATM and other proteins co-operate to reduce damage to the hair follicle caused by the regular cellular processes during hair growth and/or pigmentation and the external factor associated with the chronological aging/greying process.

## Supplementary information

Supplementary Information.
