# Supplementary material for: Stress-sensing in the human greying hair follicle: Ataxia Telangiectasia Mutated (ATM) depletion in hair bulb melanocytes in canities-prone scalp
Source: Sci Rep. 2020 Oct 30;10:18711. doi: 10.1038/s41598-020-75334-9 (PMC7603349; doi:10.1038/s41598-020-75334-9)
Supplement: Supplementary file 1 — Supplementary Information. [file 41598_2020_75334_MOESM1_ESM.pdf]

# Stress-sensing in the human greying hair follicle: Ataxia Telangiectasia Mutated (ATM) depletion in hair bulb melanocytes in canities-prone scalp.

***Stephen K. Sikkink[1], Solene Mine[2], Olga Freis[2], Louis Danoux[2], & *Desmond J. Tobin[1,3]**

[1]Centre for Skin Sciences, School of Life Sciences, University of Bradford, UK. [2]BASF Beauty Care Solutions France S.A.S., Pulnoy, France. [3]The Charles Institute of Dermatology, School of Medicine, University College Dublin, Dublin, Ireland.

***Corresponding Authors**

Dr. Stephen Sikkink. Centre for Skin Sciences, School of Life Sciences, University of Bradford, Richmond Rd. Bradford. West Yorkshire. BD7 1DP, UK. Correspondence by email to s.k.sikkink@bradford.ac.uk

Prof. Desmond J Tobin, The Charles Institute of Dermatology, University College Dublin, Dublin 4, Ireland. Correspondence by email to desmond.tobin@ucd.ie

**Supplementary Table 1. Sources of antibodies used for Immunohistochemistry.** All primary antibodies were used at a 1:100 dilution for immunohistochemistry apart from GP100 which was used at a 1:50 dilution.

| | |
|---|---|
| **Melanocyte Markers** | |
| Tyrosinase (TYR) | Abcam Mouse Monoclonal #Ab738 |
| GP100 (NKI/beteb) | Monosan Mouse Monoclonal #MON7006-1 |
| Tyrosinase related protein-1 (TRP-1) | Santa Cruz Rabbit Polyclonal #sc-25543 (H-90) |
| Dopachrome tautomerase (TRP-2) | Santa Cruz Rabbit Polyclonal #sc25544 (H-150) |
| **Apoptosis Markers** | |
| Caspase-2 (CASP-2) | BD Biosciences Monoclonal #611023 (ICH-1L) |
| Caspase-3 (CASP-3) | Abcam Rabbit Monoclonal #Ab32042 |
| Caspase-9 (CASP-9) | Abcam Rabbit Polyclonal #Ab63342 |
| BCL-2 | Santa Cruz Mouse Monoclonal #Sc-509 |
| **Cell Cycle/Senescence Markers** | |
| ATM | Abcam Rabbit Monoclonal #Ab32420 |
| p21 | Abcam Rabbit Polyclonal #Ab18209 |
| p38 | CST Rabbit Polyclonal #9212 |
| phospho-p38 (Thr180/Tyr182) | CST Rabbit Polyclonal #9211 |
| p27 | CST Rabbit Polyclonal #3686 |
| phospho-p27 (Ser10) | Santa Cruz Rabbit Polyclonal #Sc-12939 |
| p57 | CST Rabbit Polyclonal #2557 |
| phospho-p57 (Thr310) | CST Rabbit Polyclonal #2558 |
| p53 | Abcam Mouse monoclonal #Ab1101 |
| phospho-p53 (Ser15) | Abcam Rabbit Polyclonal #Ab1431 |
| **DNA Repair Markers** | |
| GADD45 | Santa Cruz Rabbit Polyclonal #sc-797 |
| 8-OHdG | Acris Mouse Monoclonal #BM2551 |
| **Anti-oxidant Enzymes** | |
| Catalase | Sigma Mouse Monoclonal #C0979 |
| Heme oxygenase-1 (HO-1) | Abcam Rabbit Monoclonal #Ab52947 |
| Superoxide dismutase (SOD-1) | Abcam Rabbit Polyclonal #Ab13498 |
| Glutathione peroxidase (GP-1) | Abcam Rabbit Polyclonal #Ab22604 |
| Glutathione reductase (GR-1) | Abcam Rabbit Polyclonal #Ab16801 |

**Supplementary figure 1. Tyrosinase staining is markedly reduced in greying hair follicles compared to GP100 and TRP-1.**

Double IF of scalp tissue with TRP-1 (green channel) & TYR (red channel) reveals a decrease in melanocyte labelling from fully pigmented to grey hair follicles. TRP-1 staining was again proportional to the melanin content observed in brightfield images (left panels; hair follicle outline is indicated with black dashed line), however by comparison TYR staining was markedly reduced even in heavily pigmented follicles. Circles in lower panels show TRP-1 negative/TYR positive staining of the last remaining pigmented melanocyte (which is still pigmented in the brightfield image) in a grey hair bulb with near complete (fully grey) canities. DAPI nuclear stain is shown in blue channel. Scale bar = 20 µm. Donor age is indicated on the right.

**Supplementary figure 2. Expression of ATM in epidermis and dermis.**

Overlays of ATM (green channel) and melanocyte marker GP100 (red channel) in two cases of adult human scalp showing examples of strong nuclear ATM expression in epidermal melanocytes. Less frequent nuclear ATM staining was occasionally detected in isolated differentiating keratinocytes within the basal layer. Similar levels of nuclear ATM staining were not detected in dermal fibroblasts, although some cytoplasmic staining was visible. Right panels show increased magnification (×2) of the epidermis. Yellow arrows indicate double-labelled melanocytes. DAPI nuclear stain is shown in blue channel. Scale bar is shown on upper images. Donor age is indicated on the right.

**Supplementary figure 3a. Caspase 2 staining in human hair follicles.**

CASP-2 (green channel) expression in two grey hair follicles from a 40-year old normal healthy female donor. Lower panels show expression in the lower IRS and connective tissue sheath in the proximal hair follicle bulb. Upper panels show CASP-2 expression in the IRS/pre-cortex in the upper hair follicle. DAPI nuclear stain is shown in blue channel. Scale bar = 20 µm.

**Supplementary figure 3b. Caspase-9 staining in human hair follicles.**

CASP-9 (green channel) expression in the bulbs of two pigmented (upper left panels) and two greying (upper right panels) hair follicles showing particularly high expression within the IRS which extended up the follicle (lower two panels). Staining was also occasionally detected within the matrix keratinocytes (white circle). Melanocytes labelled with GP100 are shown in the red channel. DAPI nuclear stain is shown in blue channel. Scale bar = 20 µm. Donor age is indicated next to each image.

**Supplementary figure 3c. GADD45 staining in human hair follicles.**

GADD45 (green channel) expression was detected in the early differentiating IRS and in the dermal papilla in hair follicles from different donors. Melanocytes labelled with GP100 are shown in the red channel. DAPI nuclear stain is shown in blue channel. Scale bar = 20 µm. Donor age is indicated next to each image.

**Supplementary figure 3d. p21 staining in human hair follicles.**

Left panel shows striking p21 expression (green channel) in terminally differentiating IRS and dermal papilla (DP) basement membrane from a 23-year old pigmented hair follicle. In comparison the right panel shows less intense IRS p21 expression with additional DP staining in an older 52-year old female. Melanocytes labelled with GP100 are shown in the red channel. DAPI nuclear stain is shown in blue channel. Scale bar = 20 µm.

**Supplementary figure 3e. Infrequent p57 and phospho-p57 staining in human hair follicles.**

p57 (left upper and lower panels) and phospho-p57 (right upper and lower panels) staining (green channel) was only detected in hair follicles from a 40-year old normal healthy female donor. p57 staining was seen within a distinct region of the ORS in a grey follicle, with expression terminating in the ORS just above the bulb region, whereas phospho-p57 expression was seen in the IRS and extended up towards the bulge region. DAPI nuclear stain is shown in blue channel. Scale bar = 20 µm.

**Supplementary figure 3f. GR-1 staining in human hair follicles**

GR-1 expression (green channel) was detected specifically in the ORS of multiple hair follicles (pigmented hair follicles from four different donors are shown). Melanocytes labelled with GP100 are shown in the red channel. DAPI nuclear stain is shown in blue channel. Scale bar = 20 µm.

**Supplementary figures 3g & 3h. HO-1 staining in human hair follicles**

Upper panels (supplementary figure 3g) show HO-1 expression (green channel) in the IRS with specific involvement of a small subset of cells within the hair follicle where IRS cornification is advanced to complete (white arrows) in hair follicles from four donors. Lower panels (supplementary figure 3h) displays two images from a transversely-(serial) sectioned scalp sample from a 50-year old female donor. Strong HO-1 cytoplasmic expression is seen in the IRS cells around the hair fibre (HF). HO-1 expression was also detected in the sebaceous gland (SG). GP100 expression of melanocytes is shown in pigmented hair follicles (red channel). DAPI nuclear stain is shown in blue channel. Scale bar is shown on images. Donor age is indicated next to each image.

**Supplementary figure 4.  Catalase expression is increased in HFMs after exposure to hydrogen peroxide (uncropped Western blots).**

Full uncropped Western blots from figure 4d. Upper image shows catalase protein expression in HFM's after treatment for 1 h with 40 µM hydrogen peroxide. CML = control untreated HFM lysate used to fill empty well. M= molecular weight marker. The catalase blot was stripped and reprobed for GAPDH (lower image) as a loading control.

**Supplementary figure 5. Total ATM protein expression is reduced by KU60019 in HFM.**

Melanocytes were treated with the ATM specific kinase inhibitor KU60019 (5 µM) in triplicate for 24 h then analysed for ATM protein expression by Western blot. Representative Western blot showing reduction of ATM protein expression in KU60019 treated samples

compared to vehicle controls (not significant by t-test). GAPDH (36 kD) was used as a loading control. Graph shows quantification of mean ATM expression (normalised to GAPDH in relative densitometry units) from Western blots. Error bars show SEM. Uncropped images of blots and size markers are also shown.

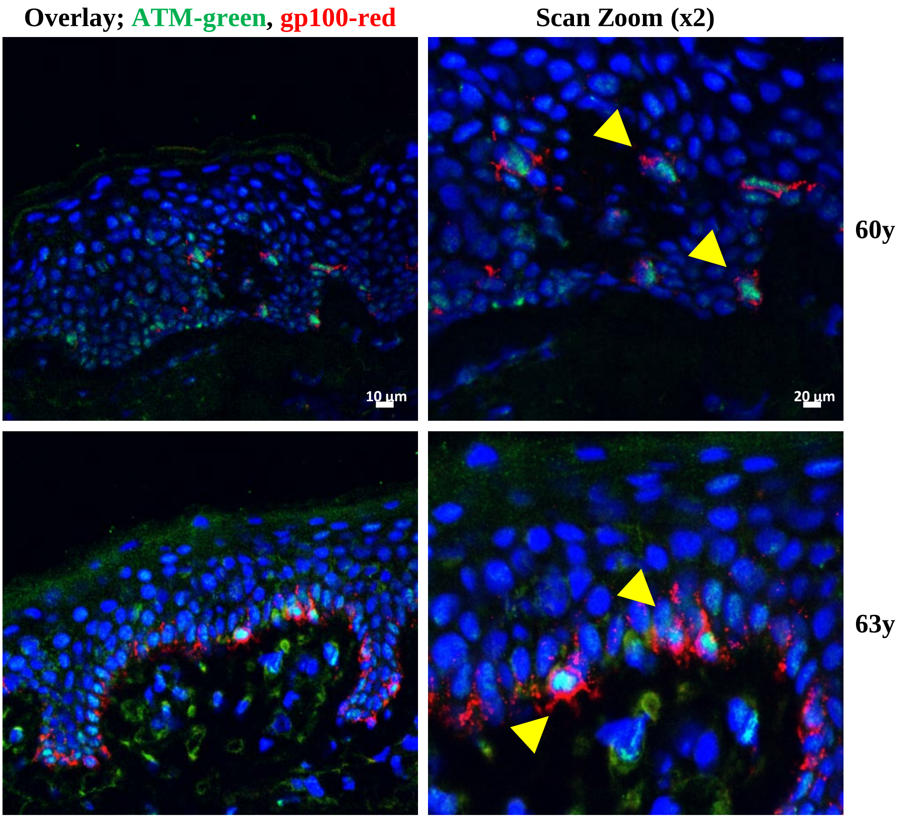

Overlay; ATM-green, gp100-red
Scan Zoom (x2)
10 µm
20 µm
60y
63y

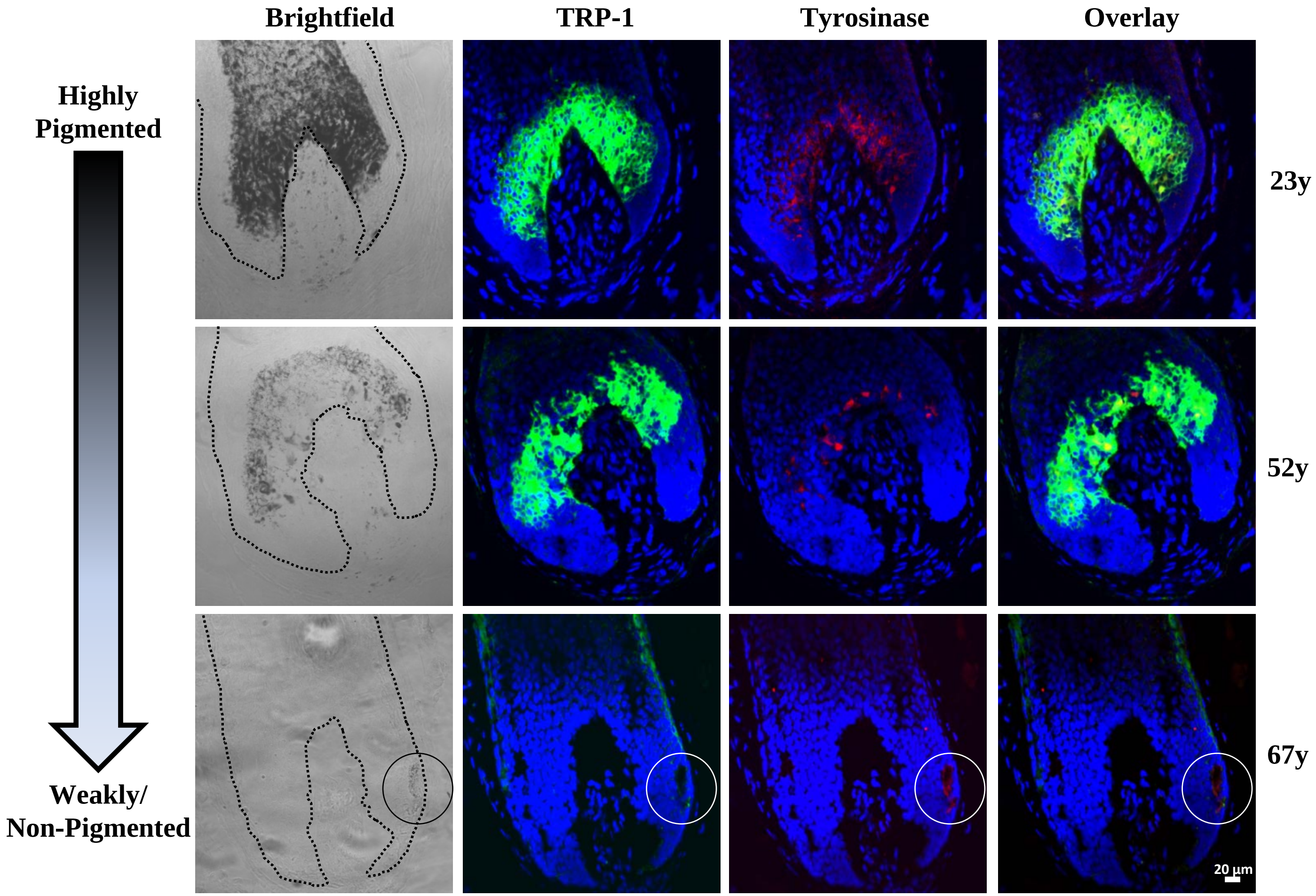
Brightfield
TRP-1
Tyrosinase
Overlay
Highly Pigmented
Weakly/ Non-Pigmented
23y
52y
67y
20 µm

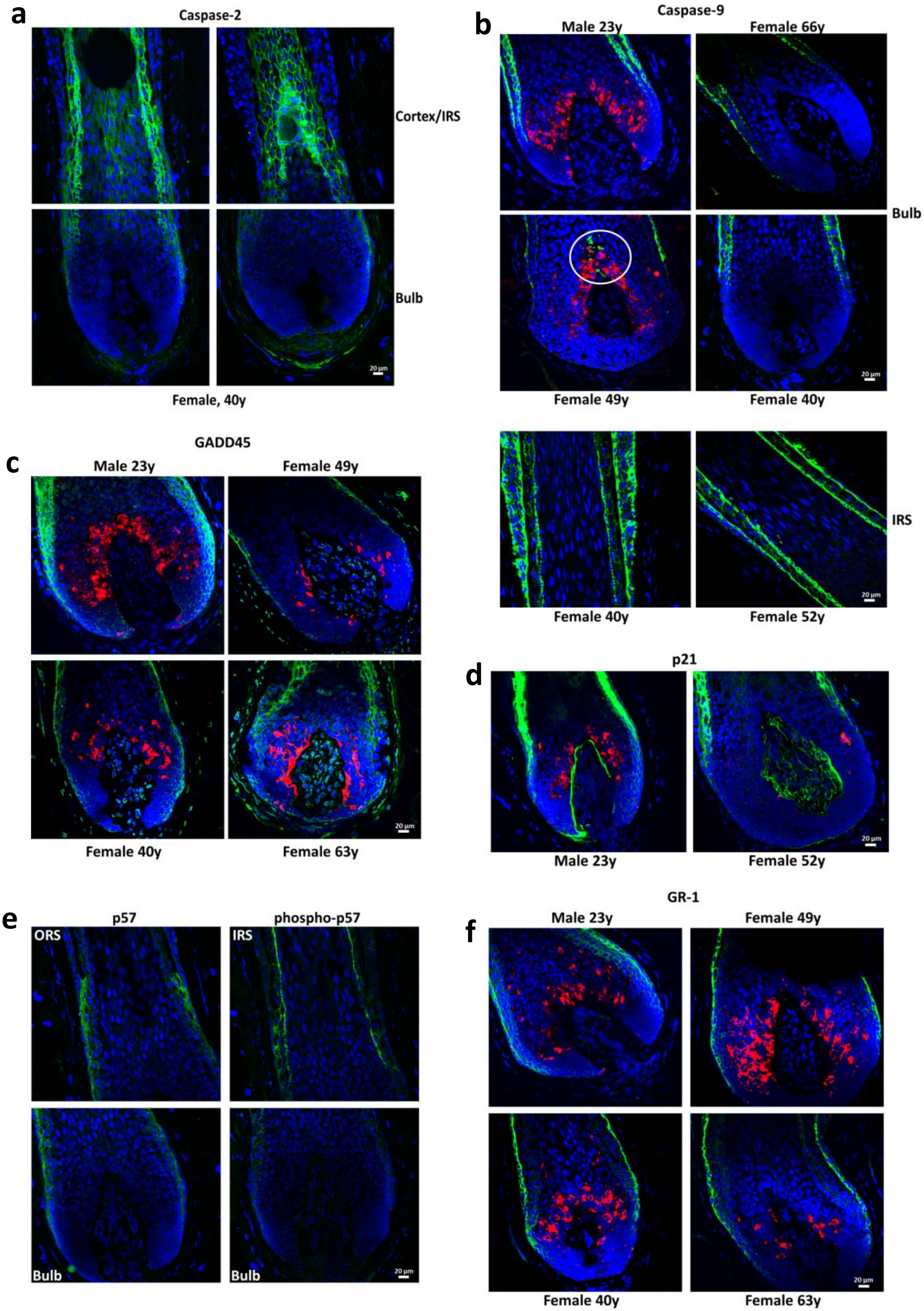
a
Caspase-2
Cortex/IRS
Bulb
20 µm
Female, 40y
b
Caspase-9
Male 23y
Female 66y
Bulb
20 µm
Female 49y
Female 40y
IRS
20 µm
Female 40y
Female 52y
c
GADD45
Male 23y
Female 49y
20 µm
Female 40y
Female 63y
d
p21
20 µm
Male 23y
Female 52y
e
p57
phospho-p57
ORS
IRS
Bulb
Bulb
20 µm
f
GR-1
Male 23y
Female 49y
20 µm
Female 40y
Female 63y

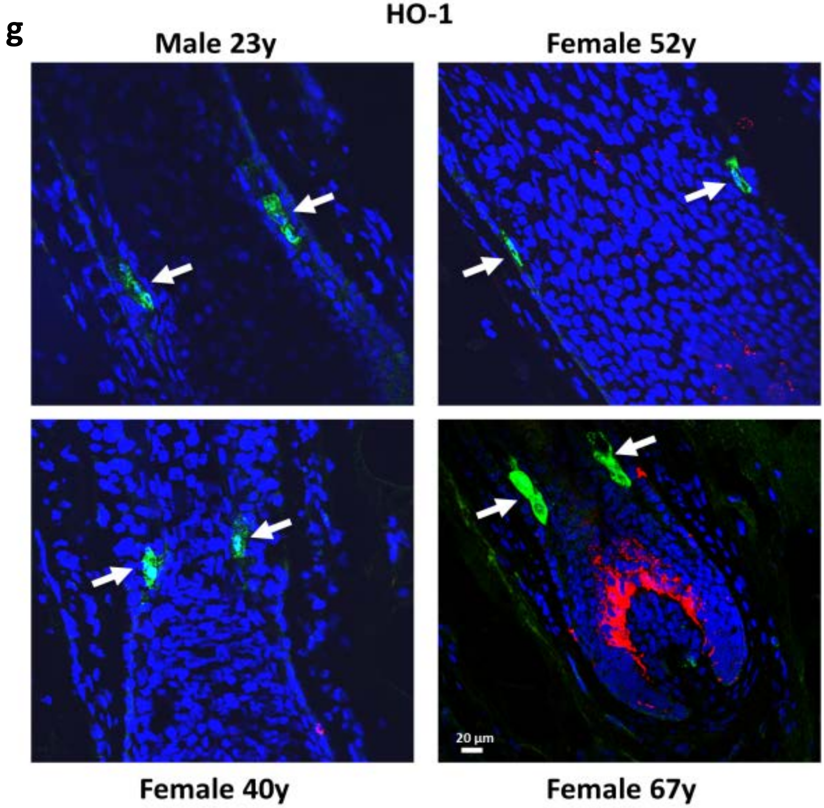
g
HO-1
Male 23y
Female 52y
20 µm
Female 40y
Female 67y

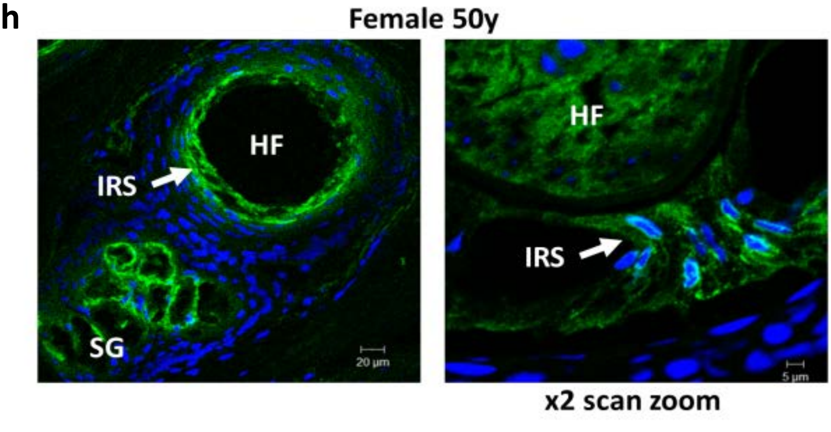
h
Female 50y
HF
IRS
SG
20 µm
HF
IRS
5 µm
x2 scan zoom

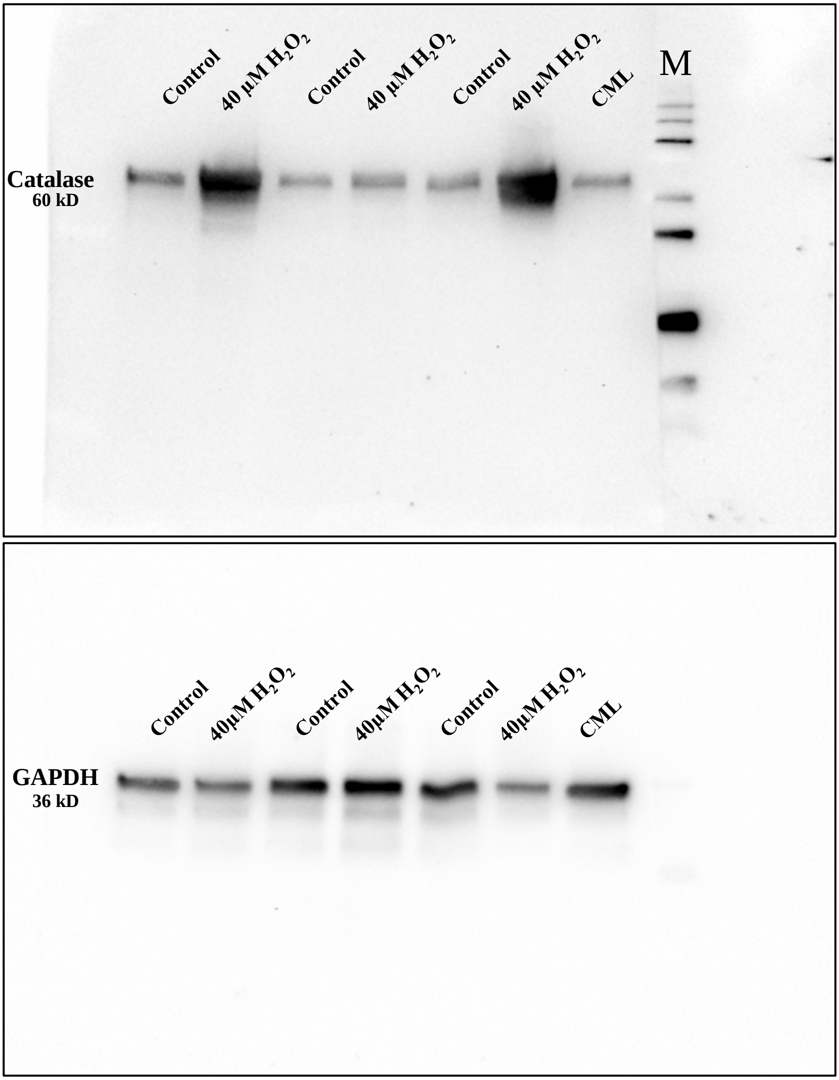
Control
40 μM H2O2
Control
40 μM H2O2
Control
40 μM H2O2
CML
M
Catalase
60 kD
Control
40μM H2O2
Control
40μM H2O2
Control
40μM H2O2
CML
GAPDH
36 kD

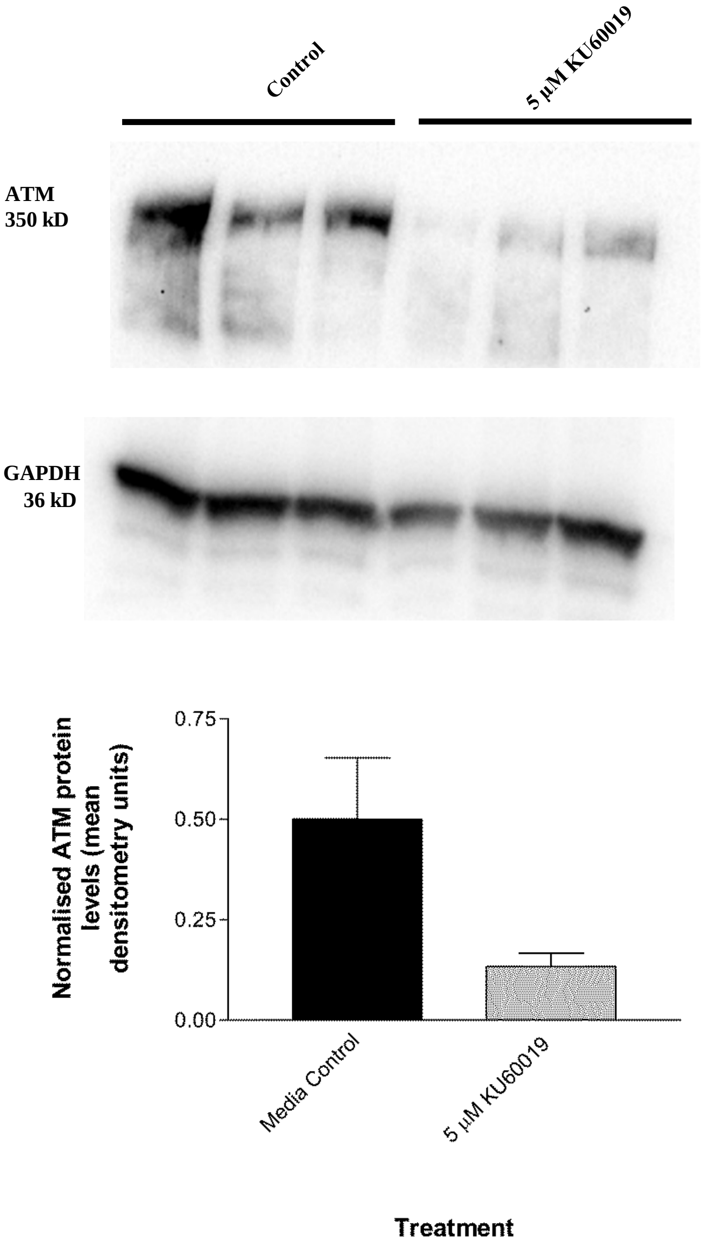

Control
5 µM KU60019
ATM
350 kD
GAPDH
36 kD
Normalised ATM protein levels (mean densitometry units)
0.75
0.50
0.25
0.00
Media Control
5 µM KU60019
Treatment

# Supplementary Figure 5 Full Western blot

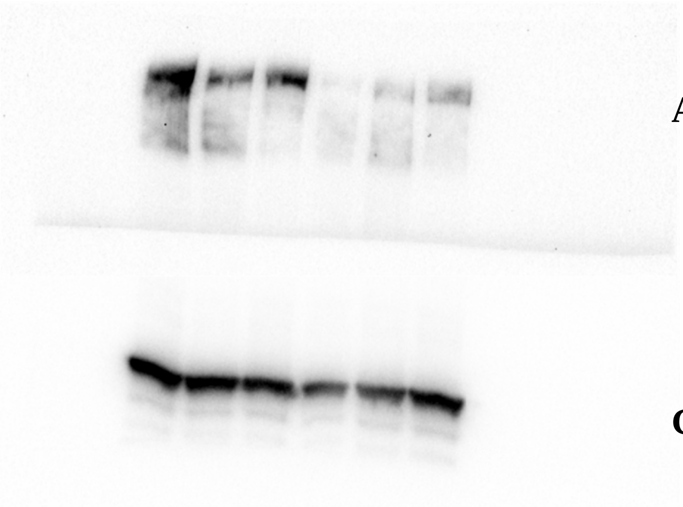

ATM blot

GAPDH blot

~350 kD

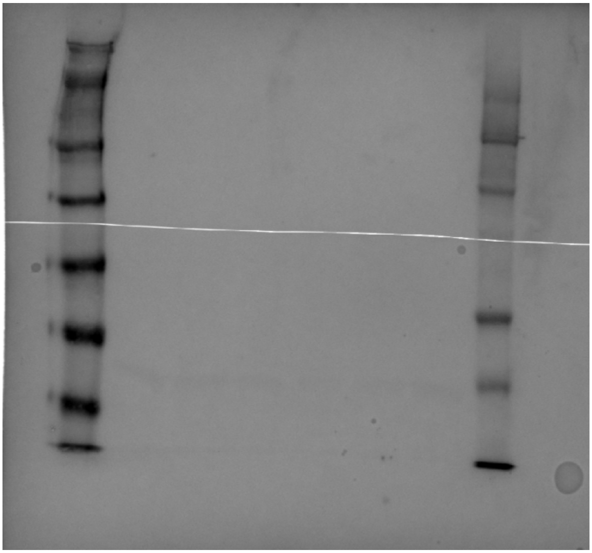

~36 kD

Size markers
